# Transmesocolic Hernia of the Ascending Colon: A Rare Cause of Small Bowel Obstruction in an Older Patient

**DOI:** 10.70352/scrj.cr.25-0293

**Published:** 2025-07-15

**Authors:** Yuki Yokota, Shumpei Okimura, Jota Mikami, Jun Kajihara, Toshihiro Kimura, Takahiko Tanigawa

**Affiliations:** Department of Surgery, Kawasaki Hospital, Kobe, Hyogo, Japan

**Keywords:** transmesocolic hernia, ascending colon, internal hernia, small bowel obstruction, older female patient

## Abstract

**INTRODUCTION:**

Transmesocolic hernia of the ascending colon is an extremely rare cause of small bowel obstruction. Due to its rarity and nonspecific clinical features, preoperative diagnosis of internal hernia is challenging.

**CASE PRESENTATION:**

We report the case of a 95-year-old female patient (body mass index: 19.5) without a history of abdominal surgery, who presented with vomiting and abdominal pain. The patient had a medical history of cerebral infarction, pneumonectomy, hypertension, hyperlipidemia, and dementia. Laboratory test results revealed leukocytosis and mild inflammation. Abdominal CT revealed closed-loop ileus on the left side of the ascending colon with localized small bowel dilatation. Chest CT indicated aspiration pneumonia. Based on these findings, a preoperative diagnosis of an internal hernia with strangulated ileus and aspiration pneumonia was made, necessitating an emergency surgery. Intraoperatively, a segment of the jejunum located 50–70 cm from the ligament of Treitz was herniated through a congenital defect in the ascending mesocolon. The ischemic jejunal bowel was resected and the mesenteric defect was closed. The operative time was 81 min with minimal blood loss. The patient experienced no surgical complications and was discharged on postoperative day 50, following treatment for aspiration pneumonia.

**CONCLUSIONS:**

Although transmesocolic hernias of the ascending colon are extremely rare, they should be considered in the differential diagnosis of small bowel obstruction, particularly in older, thin female patients without a history of abdominal surgery. Early diagnosis and timely surgical intervention are essential for achieving favorable outcomes.

## INTRODUCTION

Internal hernias are defined as the protrusion of a viscus, usually into the small intestine, through a normal or abnormal aperture within the peritoneal cavity, without any external herniation. Although rare, accounting for less than 1% of all cases of intestinal obstruction, internal hernias are clinically significant due to their potential for causing closed-loop obstruction and strangulation, which can be life-threatening if not promptly recognized and treated.^1–3)^

Among the various types of internal hernias, transmesocolic hernias and herniations of the small bowel through defects in the mesocolon are particularly uncommon. Most reported cases involve the transverse or sigmoid mesocolon, whereas defects in the ascending mesocolon are exceedingly rare.^4,5)^ The etiology of mesocolic defects can be either congenital, due to incomplete embryological fusion or resorption of the mesentery, or acquired, secondary to trauma, surgery, or inflammatory processes.^2,6)^

Congenital mesocolic defects involving the ascending colon are exceedingly rare, with very few cases documented in the literature to date.^7–9)^ These defects might remain asymptomatic until late in life and can lead to acute obstruction when the small bowel loops herniate through the defect. Notably, transmesocolic hernias might occur in patients without prior abdominal surgery or trauma, making clinical suspicion and radiographic interpretation even more critical for establishing an accurate diagnosis. The clinical manifestation of internal hernias is often nonspecific and commonly includes abdominal pain, nausea, vomiting, and signs of bowel obstruction. Contrast-enhanced CT has become the imaging modality of choice.^4,10)^

Herein, we present a rare case of small bowel internal hernia caused by a congenital defect of the ascending mesocolon in a 95-year-old woman without a history of abdominal surgery. We aim to highlight the importance of including rare types of internal hernias in the differential diagnosis of bowel obstruction, particularly in older, thin patients without a prior surgical history. In addition, we review relevant literature and discuss the clinical and radiologic characteristics, embryologic background, and surgical management of this uncommon condition.

## CASE PRESENTATION

**Fig. 1:**
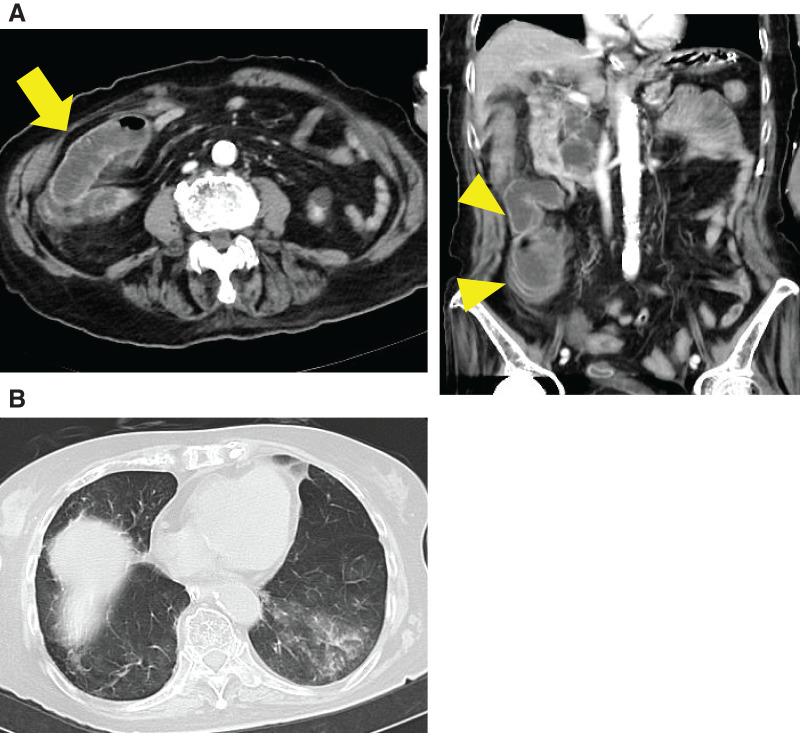
Preoperative imaging findings. (**A**) Abdominal CT scan shows dilated small bowel loops and a closed-loop obstruction (arrow, left panel). Coronal CT image highlights the herniated small bowel (arrowhead, right panel). (**B**) Chest CT demonstrates ground-glass opacity in left lung fields, suggesting aspiration pneumonia.

A 95-year-old woman (height: 142 cm; weight: 39 kg; body mass index: 19.5) presented to the emergency department with vomiting and abdominal pain. Her medical history included cerebral infarction, left pneumonectomy, hypertension, hyperlipidemia, and dementia. She reported no history of abdominal surgery. Laboratory test results revealed: white blood cell count, 19900/mm^3^; red blood cell count, 4.40 × 10^6^/μL; hemoglobin, 13.0 g/dL; platelet count, 404000/μL; serum albumin, 3.4 g/dL; aspartate aminotransferase, 10 IU/L; alanine aminotransferase, 7 IU/L; total bilirubin, 0.6 mg/dL; blood urea nitrogen, 39.8 mg/dL; creatinine, 1.20 mg/dL; and C-reactive protein, 1.9 mg/dL. These findings indicated the presence of leukocytosis and mild systemic inflammation. Abdominal CT revealed a closed-loop ileus on the left side of the ascending colon, with localized small bowel dilation (**Fig. 1A**). In addition, chest CT demonstrated ground-glass opacities in both lung fields, consistent with aspiration pneumonia (**Fig. 1B**). Based on these findings, a diagnosis of internal hernia with suspected strangulation and aspiration pneumonia was made. An emergency surgery was performed.

**Fig. 2:**
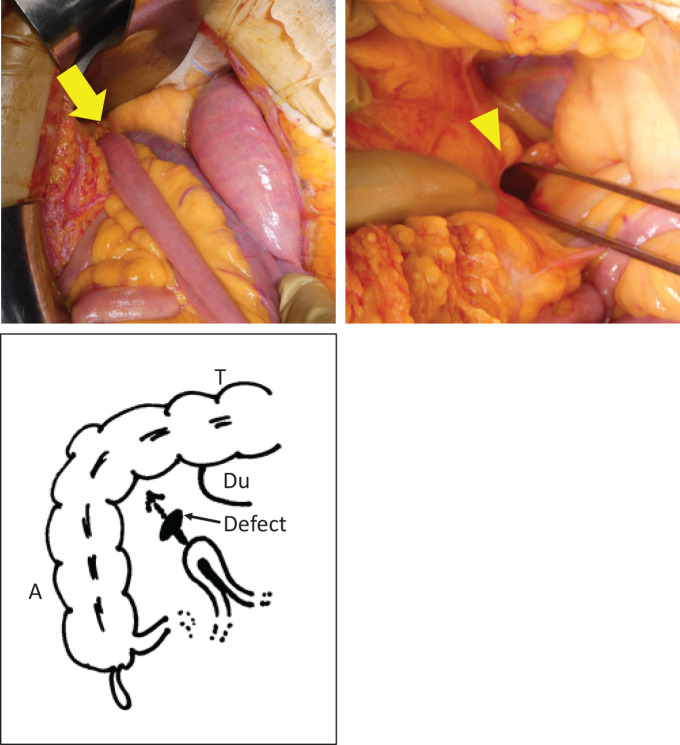
Intraoperative findings. A small bowel internal hernia was identified (arrow, upper left panel), passing through a mesenteric defect in the ascending mesocolon (arrowhead, upper right panel). Schematic of intraoperative findings (lower panel). A, ascending colon; Du, duodenum; T, transverse colon

Intraoperatively, a segment of the jejunum approximately 50–70 cm distal to the ligament of Treitz was herniated through a defect in the mesentery of the ascending colon. The affected bowel showed poor perfusion and was resected. The mesenteric defect was closed using interrupted sutures (**Fig. 2**). The operative time and pneumoperitoneum duration were 81 min. The estimated blood loss volume was 40 mL.

No postoperative complications were observed. However, due to persistent aspiration pneumonia, the patient required prolonged hospitalization and was discharged on postoperative day 50, following clinical stabilization.

## DISCUSSION

Internal hernias are rare entities, accounting for less than 1% of all cases of intestinal obstruction; they are particularly difficult to diagnose preoperatively.^1,2)^ Among them, transmesocolic hernias involving the ascending colon are exceedingly rare, with only a few cases reported in the literature.^7–9)^ The present case describes a spontaneous internal hernia of the small bowel caused by a defect in the ascending mesocolon in an older woman without a history of abdominal surgery, valuably contributing to the existing knowledge.

Transmesocolic hernias can occur due to congenital or acquired defects in the mesentery. Congenital cases are thought to arise from developmental anomalies during embryogenesis, such as failure of mesenteric fusion or resorption.^6)^ Acquired causes may include trauma, infection, inflammation, or previous surgical interventions.^2,3)^ In our case, since the patient is of advanced age, the possibility of age-related changes or abdominal trauma or infection that the patient does not remember cannot be ruled out, but in the absence of the aforementioned factors, the possibility of congenital origin cannot be definitively excluded.

The clinical manifestation of internal hernias is often nonspecific and resembles that of other causes of small bowel obstruction, including abdominal pain, nausea, vomiting, and bloating.^1,4)^ In older patients, the symptoms may be subtle or delayed, increasing the risk of progression to strangulated obstruction. Therefore, prompt diagnosis and surgical intervention are critical for avoiding bowel ischemia and reducing morbidity.

Contrast-enhanced CT plays a key role in diagnosis, with characteristic findings including clustered small bowel loops, displacement of mesenteric vessels, sac-like masses, and signs of bowel obstruction.^10)^ In the present case, contrast-enhanced CT revealed abnormal clustering of small bowel loops in the right abdomen with proximal bowel dilation, leading us to suspect an internal hernia preoperatively. Advances in CT imaging have improved diagnostic accuracy and made the preoperative identification of internal hernias more feasible.^1,10)^

Although rare, mesenteric defects in the ascending colon should be considered as a differential diagnosis in cases of small bowel obstruction in older patients without a surgical history. Reduced intra-abdominal fat and age-related changes in the connective tissue might predispose older individuals to internal herniation.^5)^

Surgical intervention is the standard treatment for transmesocolic hernias. Both open and laparoscopic approaches are feasible, involving reduction of the herniated bowel and closure of the mesenteric defect.^3,4)^ Bowel resection might be required in cases of strangulation or necrosis, highlighting the importance of early interventions.

To date, only 1 case of transmesocolic hernia of the ascending colon has been reported in the English-language literature indexed in PubMed.^7)^ Two additional cases have been described in the Japanese literature.^8,9)^ Including our case, the total number of reported cases remains limited. Although the small number of cases makes identifying specific risk factors difficult, previous reports have suggested that older, thin patients may be more susceptible to this condition (**Table 1**).

**Table 1:** Reported cases of transmesocolic hernia of the ascending colon

Author (reference)/year	Age/sex	BMI	History of urgery	Symptoms	Procedure	Outcome
Ishizaki et al.^9)^/2000	91/F	20.5	No	Abdominal pain	Primary closure of the defect	Alive
Tsukuda et al.^8)^/2006	80/M	—	No	Abdominal pain	Primary closure of the defect	Alive
Ueda et al.^7)^/2012	91/F	—	No	Vomiting	Primary closure of the defect	Alive
Our case	95/F	19.5	No	Vomiting and abdominal pain	Primary closure of the defect	Alive

BMI, body mass index; F, female; M, male

## CONCLUSIONS

Transmesocolic hernia caused by a defect in the ascending colon mesentery is an extremely rare entity. However, it should be considered in the differential diagnosis of small bowel obstruction, particularly in older, thin female patients without a history of abdominal surgery. Increased awareness and careful assessment of CT findings can lead to an early diagnosis and appropriate surgical management, ultimately improving patient outcomes.

## ACKNOWLEDGMENTS

We would like to thank Editage (www.editage.jp) for English language editing.

## DECLARATIONS

### Funding

The authors received no specific funding for this work.

### Authors’ contributions

Y.Y.: Surgical procedure, data curation, and writing—original draft preparation.

T.T.: Surgical procedure, reviewing, and editing.

S.O., J.M., J.K. and T.K.: Reviewing and editing.

All authors reviewed the text and approved the final version of the manuscript for publication.

Each author agrees to be held accountable for all aspects of the research.

### Availability of data and materials

Data will be made available on reasonable request.

### Ethics approval and consent to participate

The ethics committee of our institution approved all procedures used in this study.

### Consent for publication

Informed consent to publish has been obtained.

### Competing interests

The authors declare that they have no conflicts of interest.
